# Network analysis of narrative discourse and attention-deficit hyperactivity symptoms in adults

**DOI:** 10.1371/journal.pone.0245113

**Published:** 2021-04-07

**Authors:** Rafael Martins Coelho, Cláudia Drummond, Natália Bezerra Mota, Pilar Erthal, Gabriel Bernardes, Gabriel Lima, Raquel Molina, Felipe Kenji Sudo, Rosemary Tannock, Paulo Mattos

**Affiliations:** 1 Institute D’Or for Research and Education (IDOR), Rio de Janeiro, RJ, Brazil; 2 Department of speech and hearing pathology, Federal University of Rio de Janeiro (UFRJ), Rio de Janeiro, RJ, Brazil; 3 Physics Department, Federal University of Pernambuco (UFPE), Recife, PE, Brazil; 4 Research Institute, The Hospital for Sick Children, Toronto, Canada; 5 Institute of Psychiatry, Federal University of Rio de Janeiro (UFRJ), Rio de Janeiro, RJ, Brazil; Universtiyt of Oviedo (Spain), SPAIN

## Abstract

Previous research investigating language in attention-deficit hyperactivity disorder (ADHD) has demonstrated several deficits in many aspects. However, no previous study employed quantitative methodology providing objective measures that could be compared among different studies with diverse samples. To fill this gap, we used network analysis to investigate how ADHD symptomatology impacts narrative discourse, a complex linguistic task considered to be an ecological measure of language. Fifty-eight adults (34 females and 24 males) with a mean age of 26 years old and a mean of 17 years of educational level were administered the Adult Self-Rating Scale for ADHD symptomatology. They also completed the State-Trait Anxiety Inventory, the Beck Depression Inventory and the Urgency, Premeditation, Perseverance, Sensation Seeking Behavior Scale. Intelligence quotient was calculated. Individuals were asked to tell a story based on a wordless picture book. Speech was recorded and transcribed as an input to SpeechGraphs software. Parameters were total number of words (TNW), number of loops of one node (L1), repeated edges (RE), largest strongly connected component (LSC) and average shortest path (ASP). Verbosity was controlled. Statistical analysis was corrected for multiples comparisons and partial correlations were performed for confounding variables. After controlling for anxiety, depression, IQ, and impulsiveness ADHD symptomatology was positively correlated with L1 and negatively correlated with LSC. TNW was positively correlated with ADHD symptoms. In a subdomain analysis, both inattention and hyperactivity-impulsivity were negatively correlated with LSC. Only hyperactivity-impulsivity positively correlated with TNW and L1. Results indicated a correlation between ADHD symptoms and lower connectedness in narrative discourse (as indicated by higher L1 and lower LSC), as well as higher total number of words (TNW). Our results suggest that the higher the number of ADHD symptoms, the less connectivity among words, and a higher number of words in narrative discourse.

## Introduction

Attention-Deficit Hyperactivity Disorder is defined by the core symptoms of inattention, hyperactivity and impulsivity [1], but there is large body of evidence for deficits beyond those diagnostic features [2]. Adults with the disorder may present clinically significant impairments in tasks demanding working memory, memory span, processing speed, decision making, delay aversion to rewards, time perception, executive function, and general communication abilities [3]. As a result, an array of occupational, academic, social and domestic difficulties may arise from different sets of those deficiencies [4].

Among the phenotypic dimensions of ADHD, language problems may encompass a large set of discourse comprehension and production difficulties. A myriad of ADHD-related deficits has already been documented: poor comprehension of main plotline, ambiguous reference, event sequencing errors, incomplete clauses, discourse interruptions and embellishment. In addition—or possibly as a consequence of the aforementioned difficulties—their discourse often displays pragmatic problems like excessive speech, poor turn-taking and fail in maintaining appropriate topics in conversations. Finally, deficits were demonstrated in comprehension and elaboration of the main plot, monitorization and autocorrection of speech, online monitorization and organization of discourse and story resolution; such deficits could be a linguistic expression of executive deficits often seen in ADHD [5–12].

Narrative discourse (ND) refers to the ability of verbally reporting real or imaginary events by translating them into comprehensive structured sequences of logically-linked ideas [13,14]. It is a complex linguistic skill, which requires integration of primary language components (phonological, lexical, semantic, morphosyntax and pragmatic) with several other cognitive functions (memory, attention, planning, mental model generation and inferential production) [15–17]. Because ND assessment usually involves the reproduction of a story, it requires that the individual establishes temporal, spatial, and causal relationships among events. ND is an ecological measure of language used in daily conversations, requiring the ability to plan and organize thoughts into an expected structure easily comprehensible by the interlocutor [18–23]. Tasks addressing ND require the speaker to verbally recount an episode experienced in the present (for example, the perception of visual stimuli portrayed in scenes) or past (memory recall of events) while respecting the temporal, causal and spatial relationships among events that unfold in particular scenarios [8,24].

Previous studies investigating language in children with ADHD, reported a wide range of language problems such as: 1) less overall recall of story units, more production of ambiguous references, semantically inappropriate word substitutions, more inaccurate information and more sequence errors [6,7]; 2) misinterpretations [7]; 3) fewer verbal production, fewer utterances, fewer autocorrections then controls, and use of more words then controls to correct an error; fewer utterances related to the main plot and fewer utterances of resolution, resulting in incomplete narratives, as well with less emphasis in the main story plot then controls [8]; 4) less sustained use of the goal plan throughout their narratives, higher rates of coherence errors then control, and production of narratives resembling those produced by younger children which has less consistent use of the story goal plan [9]; 5) more difficulty producing a grammatical and fluent utterances when speakers have less syntactic flexibility, suggesting more problems with syntactic planning then controls; those deficits were seen even in adult participants who had recovered symptomatically from ADHD [11]; 6) more difficulty then controls to detect sequence errors in narrative production instructions [12]; 7) difficulties with discourse management, presupposition and narrative discourse production [8]; 8) more difficulties with global coherence in story production [8]; 9) higher rates of embellishment errors in narrative production [8] and 10) abrupt unannounced changes in topic conversations, inappropriate responses, inappropriate use of intonation, inappropriate use of pronouns and infrequent use of information by ellipsis, unintelligible rate of speech (cluttering), inappropriate loud and amount of speech, and overlapping speech [8]. All those characteristics will have consequences in discourse organization, story resolution and expected coherence in narrative production [5–9,11,12,25,26]. While functional consequences associated with linguistic abnormalities in ADHD have been increasingly recognized, instruments for assessing ND in ADHD are scarce and mostly qualitative (and therefore more prone to examiner’s biases) in children and scarce in adults [11,27,28].

The two main methods to assess language production are story retelling and conversational samples. Both methods may employ either qualitative or quantitative analyses and have given heterogeneous and sometimes discordant results according to the literature. Most studies investigating ADHD and language included samples with children and adolescents. Although there are very few previous studies investigating adult ADHD, they have either used instruments with uncertain ecological validity or qualitative analyses, which are prone to interpretation biases. The main contribution of this study is that we have used a novel quantitative approach (Speech Graph Analyses, previously used in a few psychiatric disorders) to investigate Narrative discourse, a complex linguistic skill considered to be an ecological measure [29–32].

Network analysis of discourse (also called graph analysis of speech) has been proposed as an useful method to investigate ND, providing quantitative indices of many elements, such as long and short-range recurrences of nodes (i.e., words from the story); the former being a proxy of connectedness [29–33]. Such analysis has proven to provide insights beyond language. For example, in schizophrenia graph analysis of speech demonstrated that long-range recurrence was inversely correlated with negative symptoms and impaired performance on several cognitive tests.

The present study aimed to investigate the structure of ND in adults considering their ADHD symptomatology using network analysis. For this, individuals were asked to tell a story based on a wordless picture book. We hypothesized based on previous findings that network analysis would reveal differences in ND attributes in individuals with ADHD symptoms [24]. Specifically, we predicted that higher ADHD symptomatology would be associated to a more poorly connected report (with fewer long-range and more short-range recurrences). To our knowledge, this is the first study addressing ND using this methodology.

## Materials and methods

### Sample

The study was conducted in Brazil, with Brazilian young adults subjects. All participants volunteered and signed an informed consent approved by the Ethics Committee of Copa D’Or Hospital (Submission Certification for Ethics Appreciations (CAAE) number: 38000614.1.0000.5249). Participants (n = 71) were primarily volunteers recruited among university students (graduate and undergraduate); a small number (10%) was referred by their mental health professionals aware of the study. Inclusion criteria were a) age between 18 and 40 years old, and b) current or past diagnosis of ADHD. Exclusion criteria were: a) IQ lower than 80 (WAIS-III–Wechsler Adult Intelligence Scale) [34,35]; b) current or past diagnosis of language/communication disorders, bipolar disorder or psychotic disorders; c) presence of severe anxious-depressive state (1); d) altered consciousness due to substance abuse (1); e) presence of severe sensory deficits or severe motor difficulties that precluded neuropsychological assessment; f) history of stroke, traumatic brain injury (TBI) or any known brain lesions; g) current diagnosis of uncontrolled epilepsy or delirium; h) not being a native Brazilian Portuguese speaker (1); and i) not completing the entire protocol (10). Of 71 interviewed subjects, 58 met the eligibility criteria.

Neuropsychological measure of IQ was used as screening measure for eligible criteria attendance. It was obtained using the Vocabulary and Blocks subtests of Wechsler Adult Intelligence Scale (WAIS-III) [36,37].

Individuals with known ADHD (n = 6), who were already under pharmacological treatment, were asked to undergo a 48-hour washout of psychostimulants before the assessment.

Our final sample comprised 34 females and 24 males with a mean age of 26 years old and a mean educational level of 17 years of scholarship. Their mean IQ was 119 (Table 1). Table 2 details clinical characteristics of sample.

**Table 1:** Demographic characteristics and IQ of sample.

		Count	Mean	SD
Gender	Female	34		
	Male	24		
Age			26	4
Educational level			17	3
IQ Total			119	8

Demographic characteristics and Intelligent Quotient (IQ) of sample; IQ assessed by WAIS III.

**Table 2:** Descriptive of ADHD symptoms, BDI, STAI and UPPS scales.

	Minimum	Maximum	Mean	SD
ADHD symptoms	.00	18.00	7.69	5.22
Inattention symptoms	0	9	5	3
Hyperactivity-Impulsivity symptoms	0	9	3	2
BDI	0	30	11	7
STAI—Trait	0	65	44	13
STAI—State	0	75	46	12
UPPS	0	131	101	24

ADHD current symptoms assessed by Adult ADHD Self-Report Scale (ASRS); BDI = Beck Depression Inventory for depressive symptoms dimension; STAI = State-Trait Anxiety Inventory for anxiety symptoms dimension; UPPS = Urgency, Premeditation (lack of), Perseverance (lack of), and Sensation seeking Impulsive Behavior Scale for impulsivity symptoms dimension.

### Symptomatology measures

#### ASRS

Participants were given the Adult Self Rating Scale (ASRS) in Portuguese [38] to access the current 18 ADHD symptoms pertaining to two symptom-domains: inattention and hyperactive-impulsivity; each item is scored from 0 to 3. Because there is no solid normative data for ASRS in Brazil, scores 2 (“often”) and 3 (“very often”) were considered to be positive. ADHD symptoms were used as predictors variables, and higher scores on ASRS reflect greater ADHD symptoms. ASRS has high internal consistency, with a Cronbach’s alpha coefficient of 0.84 [34].

#### State-Trait Anxiety Inventory (STAI)

The Brazilian-Portuguese version of State-Trait Anxiety Inventory is composed of two 20-item scales; with each question scored from 1 to 4; measures situational anxiety (State Subscale) and anxious trait (Trait Subscale). Shows high internal consistency estimated as Cronbach’s alpha coefficient of 0.89 [35].

#### Beck Depression Inventory (BDI)

The Brazilian-Portuguese version of Beck Depression Inventory composed of 21 questions; each item being scored on a scale value of 0 to 3; final score is a measure of depression symptom severity. BDI also shows high internal consistency estimated as Cronbach’s alpha coefficient of 0.89 [38,39].

#### UPPS

The Brazilian-Portuguese version of Urgency, Premeditation, Perseverance, Sensation Seeking Behavior Scale composed of 45 items that address four personality factors related to impulsive behavior; each item is scored on a scale from 1 to 4. UPPS provides a total score of impulsivity, and besides that, also provides subscale scores of each impulsivity subtype: lack of premeditation, urgency, sensation seeking and lack of perseverance; each one with the respective Cronbach’s alpha coefficient of 0.87 (lack of premeditation), 0.85 (urgency), 0.84 (sensation seeking), and 0.76 (lack of perseverance) [40,41].

### Narrative discourse assessment and measures

#### Narrative discourse task

The book “Frog, where are you?” [42] previously employed for assessment of healthy subjects [43–45], ADHD children [8,9,46,47]; and older adults with ADHD [24], was used in this study. It is a wordless book composed by twenty-four sequential black and white drawings telling the story of a boy who loses his frog and engages in a journey to recover it. The story unfolds in twenty-four frames evenly distributed throughout the book pages, showing events comprising a main plot (the search for the pet frog and events directly related to the aim of getting it back) and a secondary plot (peripheral series of events not necessary for comprehending the story).

The book was presented to the individual with the following instructions: “Here is a picture book. It is about a boy and his pets, a frog, and a dog. You should look at each page, and then tell me the story. I will record the story you produce. You can look through the whole book as many times as you want before we start. It is not necessary to memorize the book; you will keep it with you while you tell me the story. You should try telling the best possible story, pretending that I don’t know it”.

#### Narrative discourse evaluation and network analyses

Network analysis of discourse is the attempt to apply small-world network theory in assessment and objective description of linguistic properties. This method analyzes the networks (graphs) of transcripted discourse (language sample, in this paper, narrative discourse). As words relation in discourse is a complex system that could be represented as a network (graph), those network structures provide intuitive and useful representations for language modeling knowledge and inference [29,31,48,49]. The networks are represented by graphs composed of nodes and edges, whereas each node is a word, and each edge represents the temporal sequence between those words (links between successive words). Those graphs have attributes (speech graph attributes or SGA) that permits useful insights about language characteristics.

The analysis automatization generated the *SpeechGraphs* software, created by the same group that proposed the analysis [50]. It automatically generates network graphs representative of a transcripted language sample in a txt file. It also provides/calculates attributes from each graph. Those attributes concerns to **general characteristics** (total of Nodes (N) and Edges (E), and total of words–Word Count (WC)); **recurrence characteristics** (Repetitive Edges (RE), Parallel Edges (PE), and Loops of one, two and three nodes (L1, L2, and L3); **connectivity characteristics** (number of nodes on the Largest Connected Component (LCC), number of nodes on the Largest Strongly connected Component (LSC), and Average Total Degree (ATD)); four **global measures** (Density (D) or the amount of edges in a given graph divided by the potential number of edges given the number of nodes, Diameter (DI) or the shortest distance measured by the number of edges between the pair of nodes with the highest distance in the graph, Average Shortest Path (ASP) or the average shortest distance measured by the average number of edges between all pair of nodes in the graph, and the clustering coefficient (CC) or, considering a node, CC of this node is the estimation of how the direct linked nodes are also linked to each other, and CC of a graph is the average number of CC considering all nodes in a graph [32].

**Fig 1:**
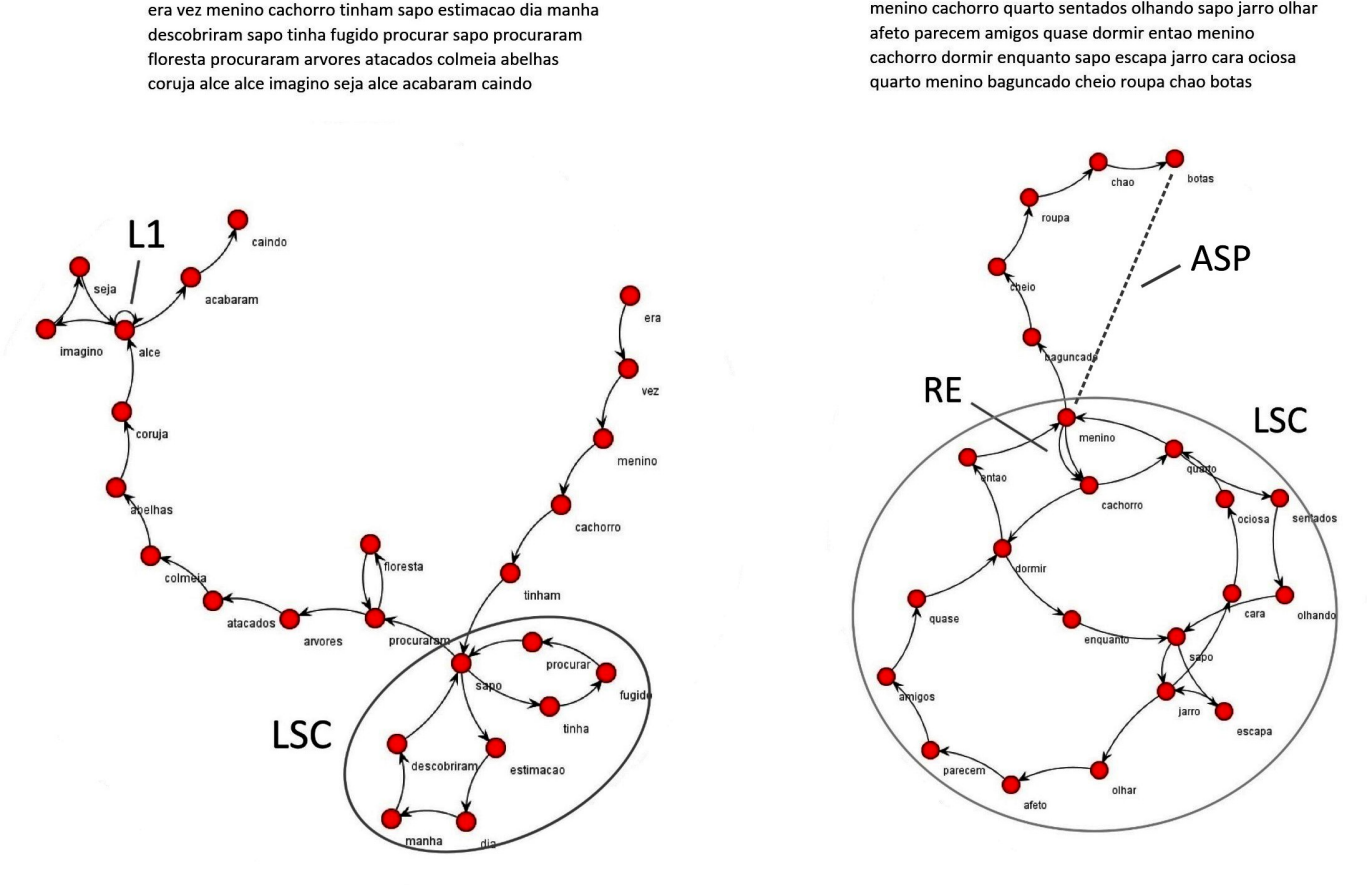
Word graphs generated by Speech Graph Analysis (SGA) software. N = node; E = edge; LSC = largest strongly connected component; L1 = number of loops of one node; RE = repeated edges; ASP = general measure of graph size.

Narratives were represented as word graphs using the *SpeechGraphs* software [32] (Fig 1). The results of SGA analysis are networks, in which each node corresponds to a word and the temporal link between two words are represented by an edge, a reliable method for speech structure investigation by non-semantic graph analysis of discourse transcripts. Preprocessing procedures text normalization like backbone speech elements examination (corresponding to subject, verb and object) and conversion to canonical elements (lexemes) [29] was not performed, since our data derived from ND elicited from a picture wordless book, which usually generates limited variations in word choice. Stop-words, comprising liaison terms with no specific meaning in oral speech (i.e.: “a”, “an”, “and”, “of”, numerals, etc.), were automatically removed from the original text. Identification of stop-words was based on the Portuguese language repository of the Stop-Words Project from Google Code Archives [51].

**Fig 2:**
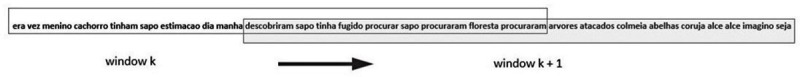
Split text method for verbosity control. Example of moving windows with 18 words length, and 50% of overlap; used to calculate mean values per graph for the different attributes.

In this present paper we chose to apply a widely used method for network analysis, where each node represents a word, different from our previous paper where each node corresponded to a different nucleus of the narrative plot. In order to control SGAs for verbosity, which has a strong influence on them according to previous findings [30,32,33,52], we used moving windows (length = 30 words, 50% overlap) (Fig 2).

We investigated elements already shown to be relevant in ND using network analysis [32]; they are depicted in Fig 1. SGAs were used as predicted variables. Total Number of Words (TNW) refers to the total number of words used in the ND. The number of loops of one node (L1) and repeated edges (RE) are measures of short-range recurrences (repetition of words, or words pairs, e.g.: short repetitions). The largest strongly connected component (LSC) is a measure of long-range recurrences (repetition of sentences or phrases with many words). The average shortest path (ASP) is a general measure of graph size.

### Statistical analysis

All the statistical analysis used the SPSS software. Normality and variance homogeneity were assessed by the Kolmogorov-Smirnov test. Spearman correlations were calculated for SG attributes (RE, L1, LSC and ASP) and ADHD symptoms assessed by ASRS scale. Partial correlation to control for confounding factors were performed with SPSS software. Statistical analysis was corrected for multiples comparisons (Bonferroni’s Correction; p = .0125).

## Results

**Fig 3:**
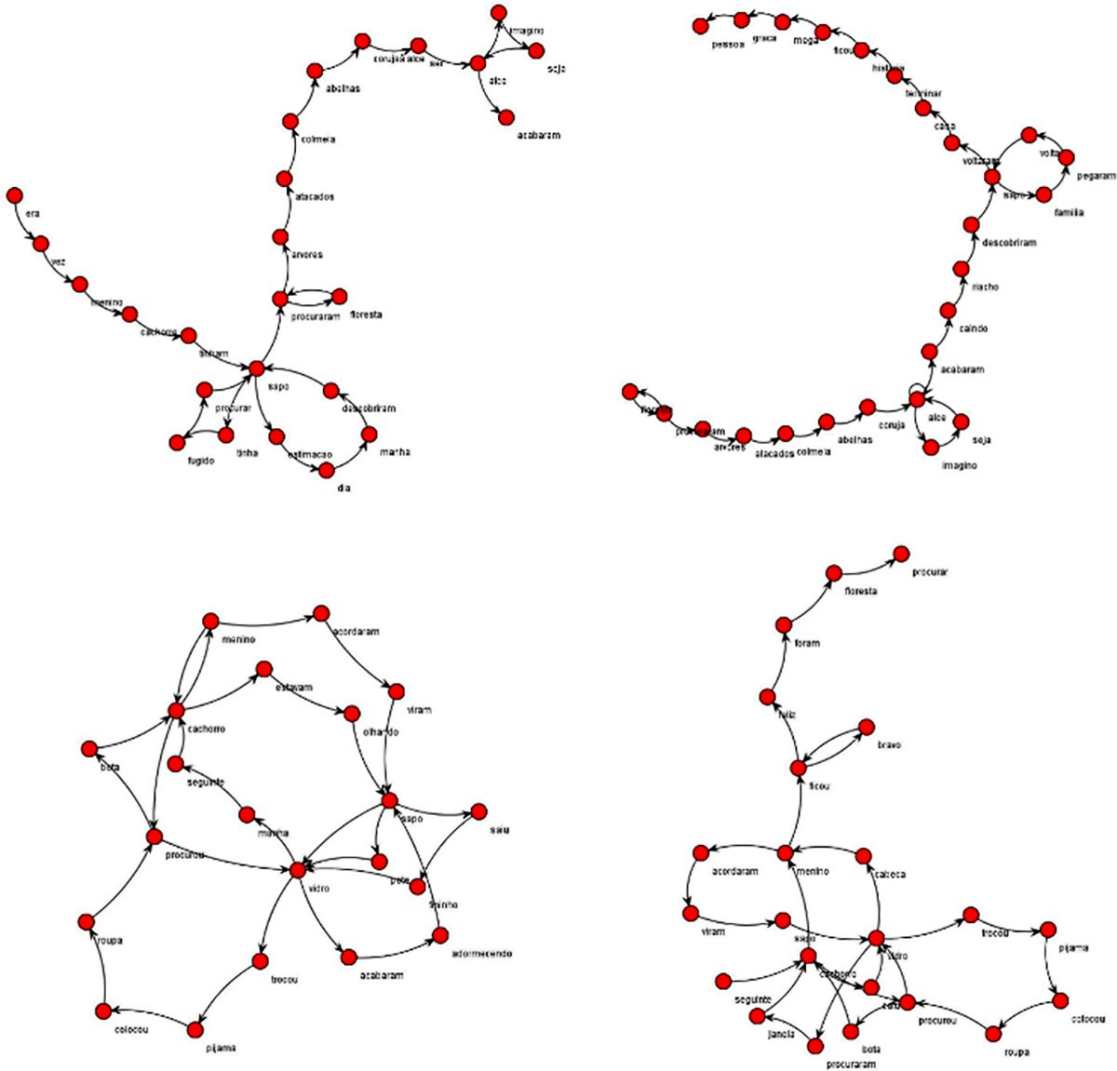
Examples of networks graphs. Examples of individual graphs from subjects with low (top pair; no ADHD symptoms) and high (bottom pair; 18 ADHD symptoms) ADHD symptoms.

Table 3 shows the correlation between current ADHD symptoms and SGA parameters. ADHD current symptoms was positively correlated with loops of one node (L1) and negatively correlated with the largest strongly connected component (LSC); a visual depiction of these findings is seen on Fig 3. In a subdomain analysis of ADHD symptoms, both current inattention and hyperactivity-impulsivity symptoms positively correlated with L1, but only Hyperactivity-impulsivity symptoms had a negative correlation with LSC. Total number of words (TNW) was positively correlated with Hyperactivity-Impulsivity. We found no correlations with ADHD and repeated edges (RE) or graph size (ASP).

**Table 3:** Correlations between TNW, RE, L1, LSC, ASP and ADHD symptoms.

	ADHD symptoms		Inattention		Hyperactivity-Impulsivity	
	*r*	*p*	*r*	*p*	*r*	*p*
**TNW**	.334	.010^a^	.251	.057	.375	.004^a^
**RE**	.090	.501	.052	.698	.054	.687
**L1**	.403	.002^a^	.316	.016^b^	.425	.001^a^
**LSC**	-.315	.016^b^	-.230	.082	-.319	.015^b^
**ASP**	.164	.220	.166	.212	.123	.358

TNW = Total Number of Words for each narrative; RE = Repetitive Edges; L1 = Loop of one Node; LSC = Largest Strongly connected Component; ASP = Average Shortest Path.

^a^Correlation after Bonferroni correction for multiple variables.

^b^Correlation at .05 significance level.

When the correlation between ADHD and SGA parameters were controlled for IQ, educational level, UPPS, BDI and STAI, the total number of words (TNW), loops of one node (L1) and largest strongly connected component (LSC) persisted in positive correlation with ADHD symptoms (Table 4). L1 positively correlated with hyperactivity-impulsivity symptoms only. LSC had negative correlation with ADHD symptoms, as well as inattention and hyperactivity-impulsivity symptoms. Repeated edges (RE) and graph size (ASP) persisted without significative correlations.

**Table 4:** Correlations between TNW, RE, L1, LSC, ASP and ADHD symptoms controlled for Age, IQ, educational level, BDI, STAI and UPPS.

	ADHD symptoms		Inattention		Hyperactivity-Impulsivity	
	*r*	*p*	*r*	*p*	*r*	*p*
**TNW**	.352	.019^b^	.296	.051	.345	.022^b^
**RE**	-.006	.970	-.012	.941	.003	.987
**L1**	.376	.012^a^	.220	.151	.498	.001^a^
**LSC**	-.434	.003^a^	-.379	.011^a^	-.409	.006^a^
**ASP**	.164	.287	.180	.243	.107	.490

TNW = Total Number of Words for each narrative; RE = Repetitive Edges; L1 = Loop of one Node; LSC = Largest Strongly connected Component; ASP = Average Shortest Path.

^a^Correlation with significance correlations after Bonferroni correction.

^b^Correlation with significance at .05 level.

## Discussion

In this study, the first to address ADHD and narrative discourse in young adults using network analysis, we provided a different framework to investigate ADHD-related language problems previously demonstrated in different languages across the world. Our findings from this quantitative methodology provide strong support for previous research in the field, however using mainly qualitative methodologies which strongly rely on the examiners’ expertise and may vary depending on the tasks employed for language assessment. Our study may provide the basis for more studies using this strategy in ADHD.

The small sample size should be considered a main limitation to this study, as well the IQ and educational level of the sample. Further studies should investigate narrative discourse in larger samples of subjects. Besides that would try to correlate with neuropsychological variables (as working memory and executive function). Notwithstanding, we used a task addressing an ecological language measure—narrative discourse (ND). The use of network analysis of narrative discourse (speech graph analysis) allowed structural quantification of language characteristics of young adults with objective and unbiased measures. We chose parameters theoretically and functionally important for narrative discourse analysis, such as attributes of short-range and long-range recurrences from SGA graph networks [30–33,52].

As we hypothesized, ADHD symptoms positively correlated with short-range recurrence (loops of one node, L1) and the opposite was found for long-range recurrence (largest strongly connected component, LSC), where a negative correlation with ADHD symptoms was found. ADHD symptomatology was associated to narrative discourses with less connectivity among words (lower LSC); our results are in accordance to a large study with children where poor language skills have been associated higher inattention or hyperactivity symptoms in primary school [52]. A genome-wide study on ADHD identified risk loci located in FOXP2 gene, involved in neural mechanisms mediating the development of speech and learning [53].

Those results suggest that the more ADHD symptoms the individual has, the more he presents short-range recurrences and fewer long-range recurrences. In typical children, less long-range recurrences (i.e., connectedness) correlated with lower IQ and lower verbal memory as well as worse performance on tasks addressing theory of mind [52,54]; increased connectedness has been associated with higher reading abilities and higher educational levels [29,52]. To date, there are no studies addressing the clinical correlates of lower connectedness in ADHD.

Because ADHD may be associated with comorbid anxious and depressive disorders [55] we controlled for those parameters; correlations persisted. In addition, because impulsivity, a hallmark of ADHD, could potentially contribute to a worse performance in narrative discourse, we have also controlled for this aspect, however obtaining similar results.

Total number of words was positively correlated with Hyperactivity-Impulsivity. Of note, this result is in accordance to DSM-5 criterion “talks excessively”. Our finding may add further understanding of previous descriptions of verbosity in adult ADHD discourse, however using a different methodology [8] ADHD associated deficits in oral narrative could be due to deficits in executive functioning (including working memory), commonly seen in ADHD. Working memory impacts the ability to organize the narrative production, maintaining the principal story plot. Executive function deficits could also lead to excessive digressions from main plot; attempts to correct or adjust an utterance already in course may lead to production of more speech, but without incremental quality or relevant information. Although our study design does not allow to draw conclusions on this, higher values for L1 (an attribute of short-range recurrence) and lower LSC (an attribute of long-range recurrence) could potentially be secondary to this behavior. Another way to interpret the higher number of short-range recurrences would be a tendency to produce short utterances (like false starts, hesitations, pauses), while the individual gains time to organize the next language structure of the topic, in order to overcome the working memory deficit. Again, our study design did not allow us to investigate those aspects; further studies will be necessary to clarify this association.

In summary, our study has demonstrated that ADHD symptoms are associated with narrative discourse problems, in particular the degree of connectedness and number of words used to convey the story. From a clinical perspective, our results suggest that whenever individuals with ADHD symptomatology are requested to narrate a story based on visual stimuli, they are more verbose, repeat more words or words pairs and produce a discourse with less cohesion among the words they have chosen to tell the plot.

Of note, in a study employing different tasks addressing ND in adults with mild dementia, it was shown that the same profile of impairment was seen across all modalities (with and without visual stimuli) [56]. There are no such studies on ADHD, but it seems reasonable to presume that the same language impairment would be present, at various levels, when ADHD patients are asked to verbally report current or past facts. One could expect ADHD individuals to portray a less cohesive and more verbose speech in clinical interviews.

## Supporting information

S1 File (XLSX)
